# pH Regulation and Tissue Coordination Pathways Promote Calcium Carbonate Bioerosion by Excavating Sponges

**DOI:** 10.1038/s41598-018-36702-8

**Published:** 2019-01-24

**Authors:** Alice E. Webb, Shirley A. Pomponi, Fleur C. van Duyl, Gert-Jan Reichart, Lennart J. de Nooijer

**Affiliations:** 1 NIOZ Royal Netherlands Institute for Sea Research, and Utrecht University, Ocean Systems, P.O. Box 59, 1790 AB Den Burg, Texel The Netherlands; 2 Harbor Branch Oceanographic Institute, Florida Atlantic University, 5600 US 1 North, Fort Pierce, FL 34946 USA; 3 NIOZ Royal Netherlands Institute for Sea Research, and Utrecht University, Marine Microbiology and Biogeochemistry, P.O. Box 59, 1790 AB Den Burg, Texel The Netherlands; 4 Faculty of Geosciences, Utrecht University, Utrecht, The Netherlands

## Abstract

Coral reefs are threatened by a multitude of environmental and biotic influences. Among these, excavating sponges raise particular concern since they bore into coral skeleton forming extensive cavities which lead to weakening and loss of reef structures. Sponge bioerosion is achieved by a combination of chemical dissolution and mechanical chip removal and ocean acidification has been shown to accelerate bioerosion rates. However, despite the ecological relevance of sponge bioerosion, the exact chemical conditions in which dissolution takes place and how chips are removed remain elusive. Using fluorescence microscopy, we show that intracellular pH is lower at etching sites compared to ambient seawater and the sponge’s tissue. This is realised through the extension of filopodia filled with low intracellular pH vesicles suggesting that protons are actively transported into this microenvironment to promote CaCO_3_ dissolution. Furthermore, fusiform myocyte-like cells forming reticulated pathways were localised at the interface between calcite and sponge. Such cells may be used by sponges to contract a conductive pathway to remove chips possibly instigated by excess Ca^2+^ at the boring site. The mechanism underlying CaCO_3_ dissolution by sponges provides new insight into how environmental conditions can enhance dissolution and improves predictions of future rates of coral dissolution due to sponge activity.

## Introduction

Boring sponges excavate and grow into calcium carbonate substrates with their activity leading to significant marine carbonate erosion^1–4^. In coral reef environments, bioerosion leads to significant loss of reef structures and may tip the balance between production and erosion in favour of the latter^5,6^.

Bioerosion by excavating sponges is achieved by a combination of chemical dissolution and mechanical chip removal^7–9^. It is hypothesised that specialised cells are able to reduce saturation state with respect to aragonite at the sponge-coral interface, resulting in dissolution of the coral’s skeleton^10^. Since the etching interface is not directly accessible for observations, investigating the underlying mechanisms by which these sponges dissolve CaCO_3_ has proven to be challenging. Recent results showed that boring rates increase with higher seawater *p*CO_2_^11–16^, suggesting that a reduced ambient saturation state directly lowers the energetic costs for the sponge to lower the saturation state in which the aragonite skeleton is dissolved. Despite the expected increase of sponge bioerosion impact on coral reefs with on-going ocean acidification, the mechanistic link between environmental conditions and bioerosion rates remains unknown.

Production of acids is thought to be ultimately responsible for CaCO_3_ dissolution^17–19^. Hatch^10^ suggested that excavation by sponges involves a localized modification of the CaCO_3_ solubility equilibrium. Furthermore, Hatch^10^ provided evidence for the presence of the enzyme carbonic anhydrase at the sponge-coral interface, which could facilitate the transport of H^+^ across membranes or provide an optimum pH for other chemical processes involving, for instance, calcium chelators. Sullivan and Faulkner^20^ demonstrated the presence of such calcium chelators in *Siphonodictyon coralliphagum*^21^ and described the dissolution process as a cycle in which calcium chelators release protons in exchange for calcium ions at the etching site and release calcium ions to the water outside the sponge in exchange for protons. This proposed pathway implies that a reduction of pH at the sponge-substrate interface would form the basis of sponge chemical erosion. However, a direct characterization of the conditions during aragonite dissolution is necessary to confirm that this mechanism is adopted by excavating sponges. Here we investigate the cell physiological and chemical conditions at the dissolution interface of the sponge *Cliona varians*^22^ by applying a pH fluorescent probe and subsequent visualization of pH at the etching site with fluorescence microscopy.

## Results and Discussion

**Figure 1:**
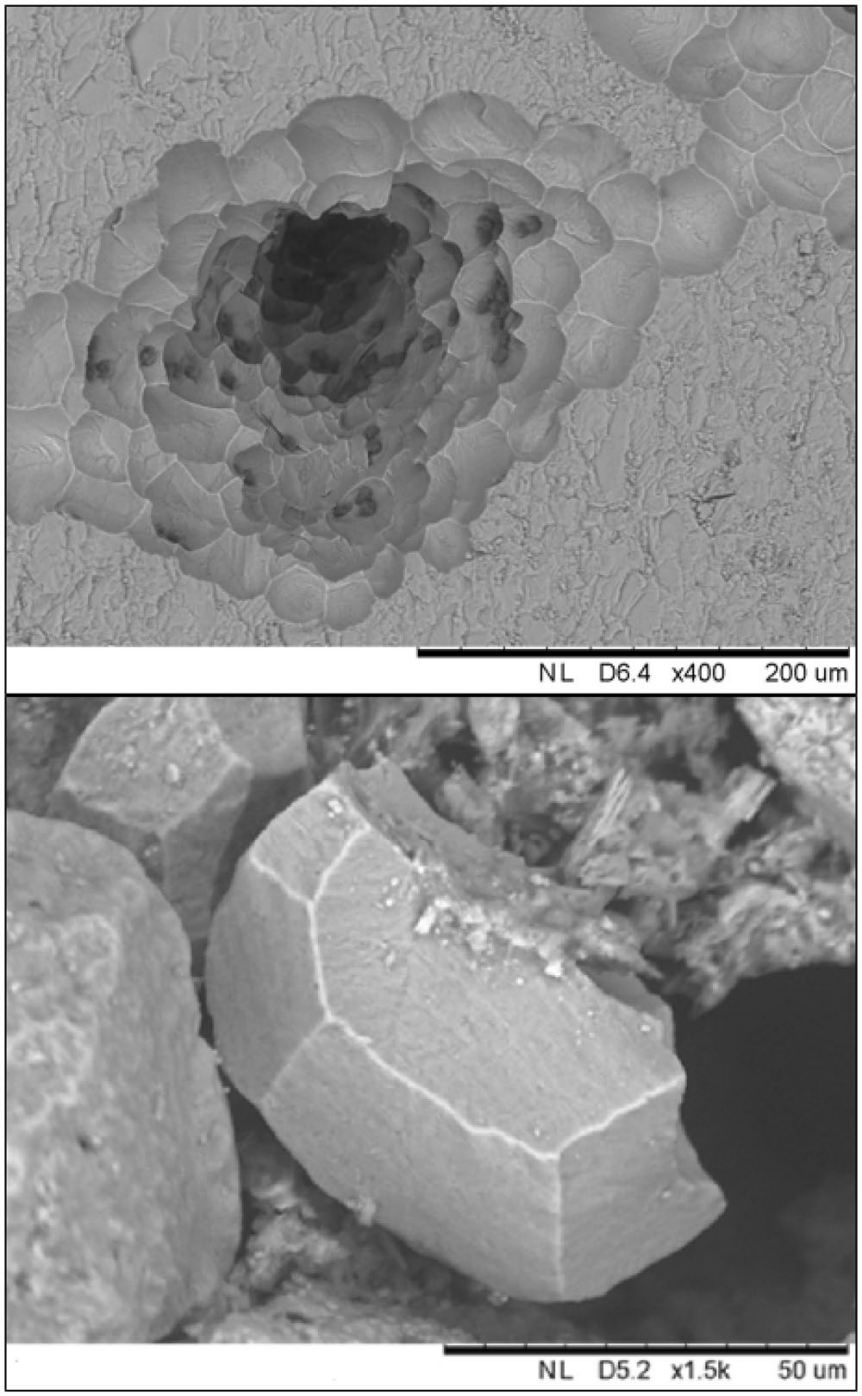
Scanning electron microscope images of calcite excavated by *C*. *varians*. The top panel depicts an excavated channel lined with characteristic pits that remain after the chips are removed; the bottom panel shows a chip with its distinctive orthogonal carved shape.

**Figure 2:**
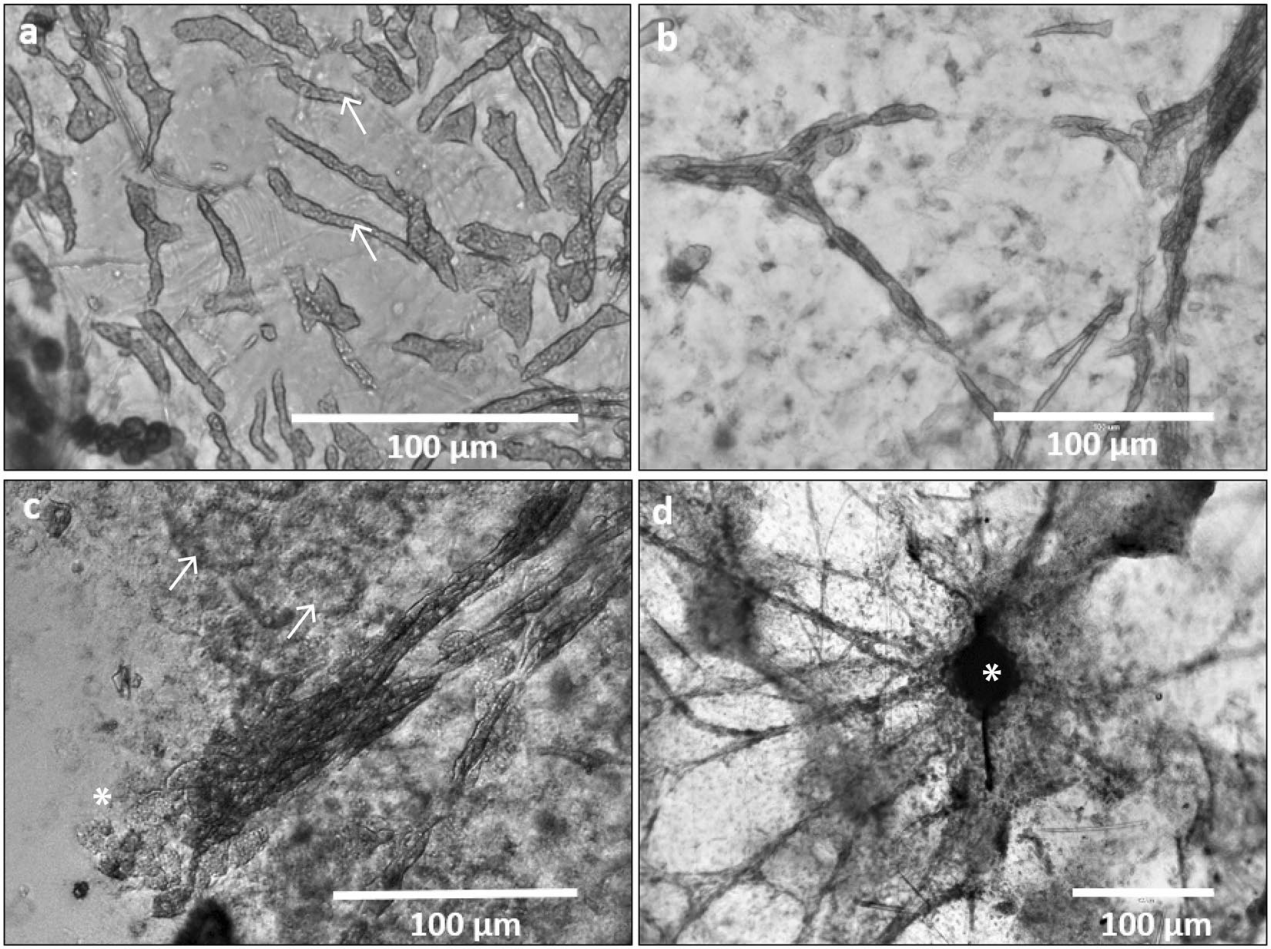
Transmitted light images of cells of *C*. *varians* organised in bundles and networks responsible for chip transport. (**a**) Elongated cells with vesicular cytoplasm merge into muscle-like pathways (**b**,**c**,**d**) spreading from pits where excavation happens. Note etching cells (*) in the lower left of (**c**) in contact with the elongated cell network and choanocyte chambers (arrows) in the upper part of (**c**). In (**d**), the fibre-like network is organised around a boring pit (*).

**Figure 3:**
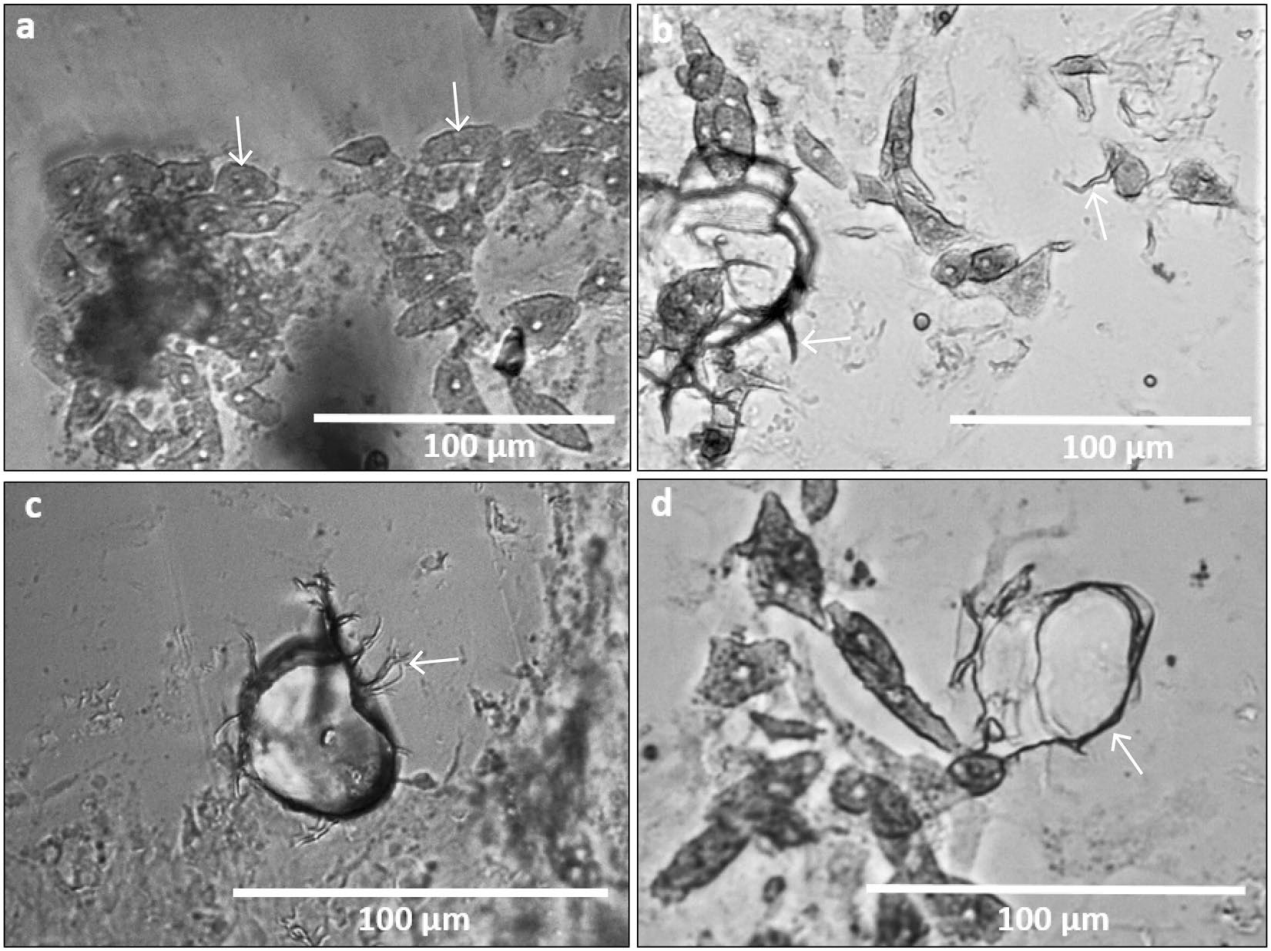
Transmitted light images of *C*. *varians* etching cells (**a**, arrows) and accompanying filopodia (**b**,**c**,**d**). (**a**) Club-like etching cells (arrows) with prominent nuclei and vesicular cytoplasm. The cells are located around two initial boring pits. (**b**) On the right, a filopodium is extruding from an etching cell (arrow). On the left, the filopodia network is developing and outlines of chips can be distinguished. (**c**) Formation of a chip by extending filopodia (arrow). (**d**) Filopodial extensions emanating from an etching cells and merging to form cytoplasmic sheets engulfing a calcite chip.

Iceland spar crystals containing tissue of *C*. *varians* that grew into the calcite were carefully detached from the “mother sponge”. This exposed a complex network of fibre-like interconnections as well as several circular perforations of the surface crystal face, filled with living tissue. Scanning electron microscopy exposed clear signs of bioerosion and calcite chips within the tissue (Fig. 1). Transmitted light microscopy revealed two dominant cell types (Figs 2 and 3) with vesicular cytoplasm likely playing key roles in bioerosion. These two cell types have a distinct morphology and localization, which reflects their function and will be subsequently discussed in light of previous descriptions on their role in chip production and removal^8,9^.

### Myocyte-like cells forming tissue coordinating pathways

At locations adjoining excavating activity, elongated fusiform cells, measuring between 25 to 80 µm in length and 2 to 5 µm in diameter, with a relatively vesicular cytoplasm (Fig. 2a), are found forming bundles that resemble pathway connections (Fig. 2b,c). Their overall appearance (e.g., Fig. 2c) resembles the organization of vertebrate muscle fibres, and hence may represent the mobile fusiform collencytes observed by Rützler and Rieger^9^. We suggest that these cells may be actinocytes/myocytes^23–25^ capable of directed transport of the chips formed in the dissolution pits through coordinated tissue contraction, although such cells have not yet been identified in excavating sponges. It should be noted that Grant^26^ created the new genus *Cliona* in reference to the high frequency of contractions of *Cliona celata*. Furthermore, De Ceccatty^27^ describes a comparable type of fixed tissue coordination pathway in the sponges *Tethya*, *Hippospongia*, *and Euspongia* that is formed through connections and bundles between muscle-like cells from the same unit structure. De Ceccatty^27^ argues that this system is based on the capacity of cells to construct a structurally defined pathway in order to promote direct exchanges from one cell to another. In the same species, electron microscopy images illustrate tight membrane appositions between adjacent cells and transfer of vesicles from cell to cell^28^.

The exclusive organization of the *C*. *varians* cells into bundles connecting the boring pits with the rest of the sponge (Fig. 2d) indicates that they are instrumental to the transport of chips out of the excavated pit and into an excurrent canal. The role of these cells in chip removal is almost a priori given the fact that the formed chips have to be transported away from the bottom of the boring pits (Fig. 2) and hence must pass these myocyte-like cells before entering the excurrent canal.

### Etching cells

Etching cells are the second dominant cell type present at the sponge/calcite interface. Their morphology changes to correspond with their ultimate function: the dissolution of calcium carbonate. Initially, they appear as club-like cells of archeocyte origin with a prominent nucleus and cytoplasm with a vesicular appearance (Fig. 3a). To penetrate the carbonate substrate, several thread-like filopodia originate from the cell body (Fig. 3b,c) and ramify to partly fuse again forming cytoplasmic sheets (Fig. 3b–d). The filopodia form a basket-shaped meshwork corresponding in size to the substratum chips they surround. Previous research also showed networks of thread-like pseudopodia originating from club-like cells and based on their morphology inferred that they were likely responsible for CaCO_3_ dissolution^7,9,17,19^. Many etching cells with their accompanying processes in this study were observed in a disintegrating stage. This collapse of cells has also been observed previously^8,9,29^ and appears to be linked to the penetration of the filopodial extensions into the substrate. The cell disintegration stage hence appears to be a function of filopodial growth.

Etching cells are very vesicular in appearance (Fig. 3a) which may be linked to the flocculent secretory product within the filopodia described by Rützler and Rieger^9^. This product was assumed to be contained within vesicles that would be subsequently emptied into a cytoplasmic sac. In the present study, no flocculent material inside the filopodia was observed using transmitted light microscopy but this may be due to the limited spatial resolution of our method. Fluorescence microscopy imaging, however, revealed low pH vesicles in the filopodia (see section below). The tissue composition and configuration around and inside burrowing tunnels formed by this excavating sponge is an indication of how dynamic the etching process is. It appears that etching cells are being continuously formed and discarded, filling every crack and cavity to ensure sponge growth and dissolution of the CaCO_3_ substratum.

### pH reduction at sponge/substrate interface

**Figure 4:**
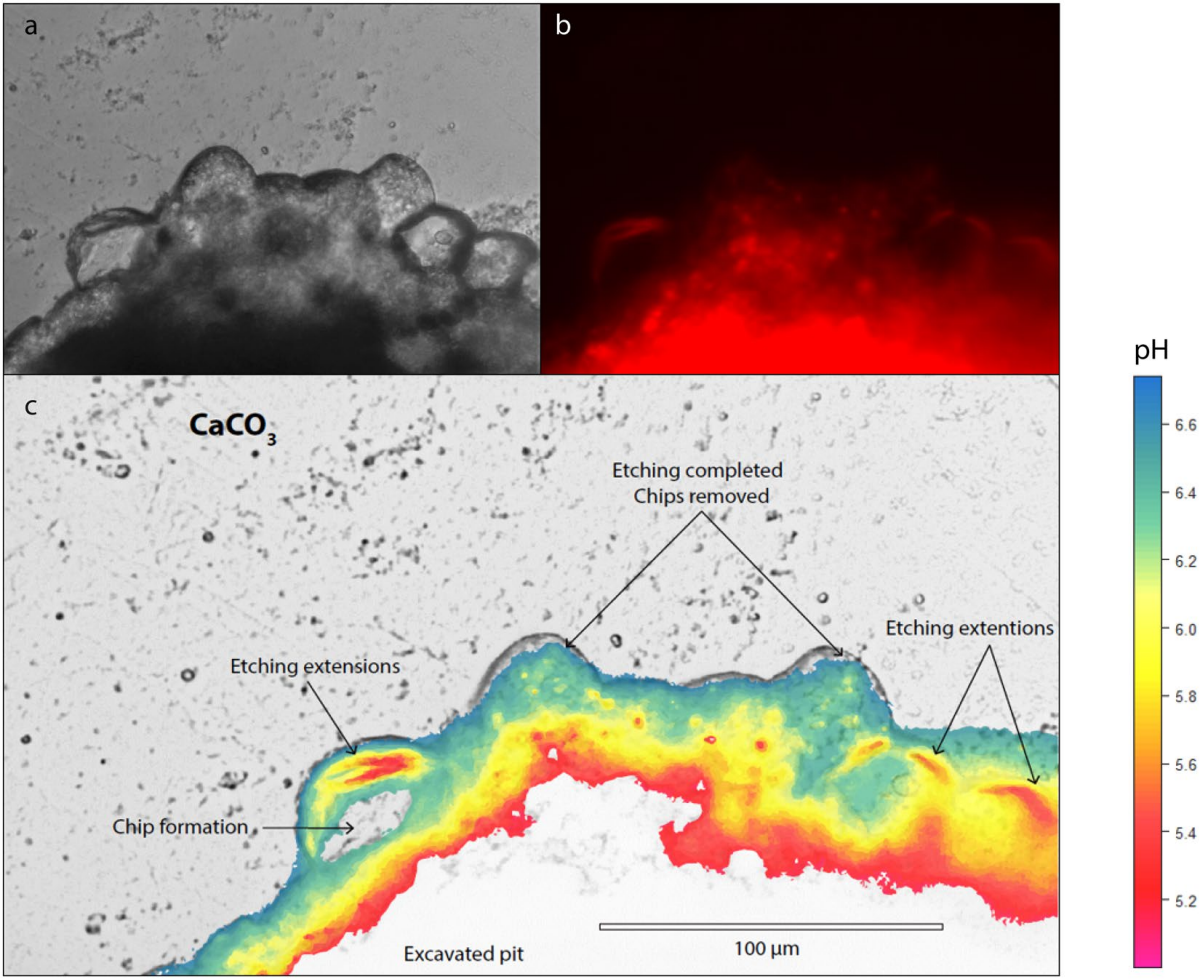
Low pH in etching filopodia in sponge excavated pit exposed after detachment of a piece of bioeroded calcite from the “mother sponge”. (**a**)Transmitted light image showing etching filopodia carving out chips on the leading edge of a bored channel in the substratum. (**b**) Z-stack imaging of the same area; red fluorescence represents pH below neutral. (**c**) Processed image using the fluorescence intensity/pH calibration (Fig. 9) showing low pH in etching filopodia that are carving chips from an excavation pit. Due to the detachment from the “mother sponge”, most tissue around the pit was removed, however, a large fraction of cells within etched crevices remained, enabling exclusive visualization of the etching filopodia. The high fluorescence levels in the centre of the pit are due to the superposition of cells causing additive fluorescence and were therefore dismissed from further analysis.

Visualization of pH inside the sponge tissue revealed that the intracellular pH within the filopodial extensions approximates 5 during carbonate dissolution, while non-bioeroding tissue approximates a pH higher than 7 (representing the upper limit of our approach). The observed lowered pH is consistent with a study using microsensors measurements where pH was found to decrease continuously as a function of tissue depth towards the substrate^30^. However, results from the latter study showed a rather modest pH change throughout the sponge tissue compared to the present study. The discrepancy between previously published and present observations may primarily reflect positioning of the microsensors as the site of dissolution is very restricted and not easily accessible. Fluorescent dyes are more suited to show changes at the actual interface. Our results suggest that *C*. *varians* locally reduce the pH at the etching site in order to dissolve calcite (Icelandic spar), we infer that the same process applies to aragonite (coral skeleton). At high magnification, it is clear that the lowest pH values are confined to filopodia of the etching cells protruding around the chips (Fig. 4). Figure 4c illustrates the active formation of 3 chips (one on the left, and two on the right). Other visible grooves are filled with cells, but no etching filopodia are discernible. We suggest that the lack of etching at these locations indicates that chip etching in these areas was completed; chips have been removed and cells only recently recolonised this space, probably reorganizing for the next phase of chip excavation.

**Figure 5:**
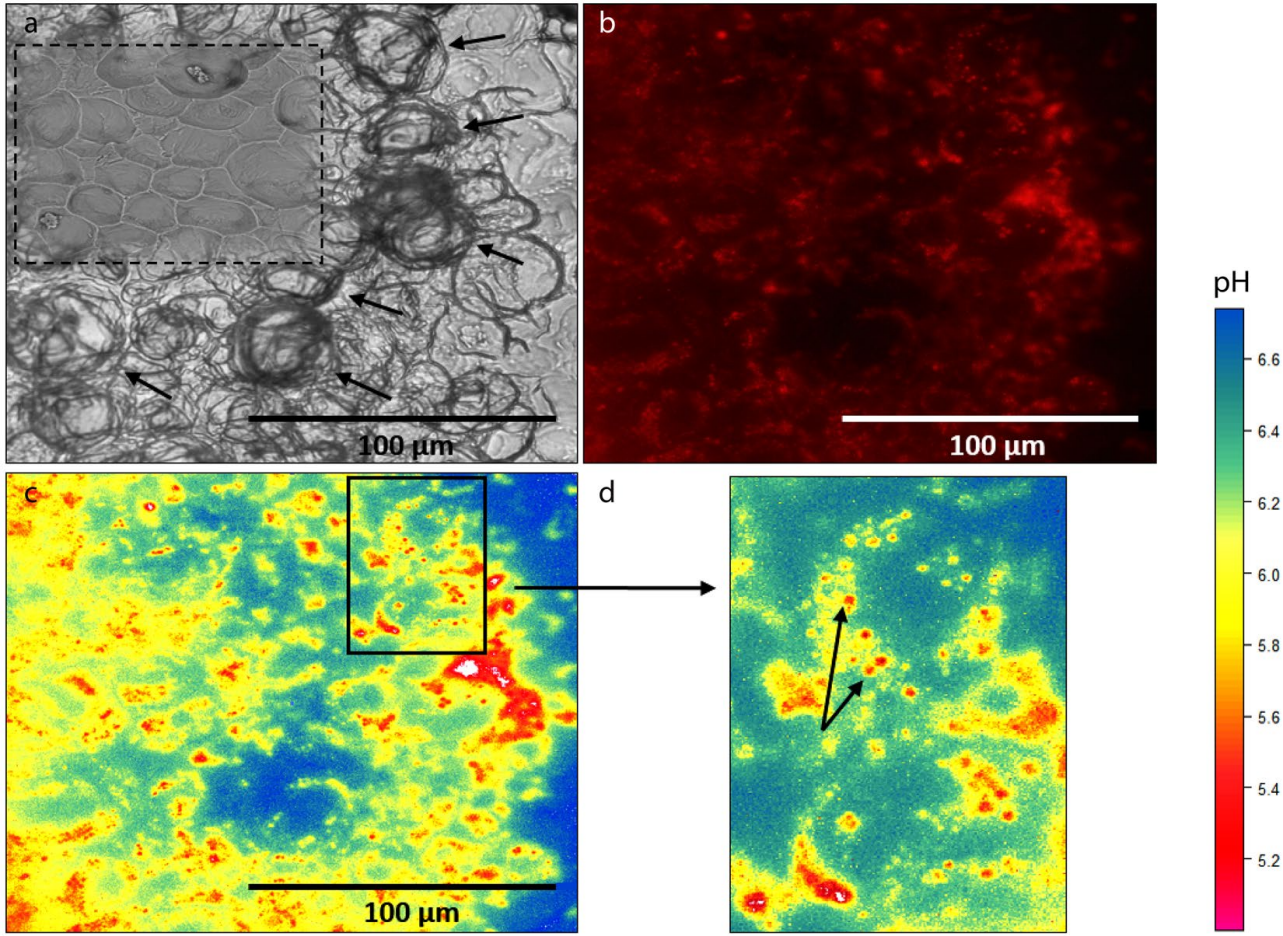
Low-pH vesicles within etching filopodia located at the bottom of an excavated pit viewed from the top. (**a**) Transmitted light image of etching filopodia indicated by black arrows and corresponding SEM image (top left) of excavated chip pits without etching filopodia (dashed square). (**b**) Z-stack imaging of the same area, where red fluorescence represents low pH, showing numerous vesicles with low pH following the outline of etching filopodia. (**c**) Processed image with pH quantification showing lower pH within vesicles with detail enlarged in (**d**).

The low pH inside the filopodia appears to reside in numerous subcellular vesicles (Fig. 5b–d) which are consistently spaced in concentric rings within the inner space of the filopodia networks. These low pH vesicles observed in the filopodia are comparable to what Rützler & Rieger^9^ refers to as a flocculent secretory product extending into the filopodia. We propose that vesicles discharge their contents in the cytoplasmic sheets, enabling the filopodia to penetrate the substrate. The absence of vesicles in Fig. 4 is likely due to vesicles having just discharged their content and dissolution neutralising the low-pH fluid. To estimate whether the vesicles within the filopodia can be fully responsible for the volume of aragonite that is dissolved around one chip, the fluorescent images and obtained intra-vesicle pH values can be combined and compared to the calculated proton budget involved in aragonite dissolution.

**Figure 6:**
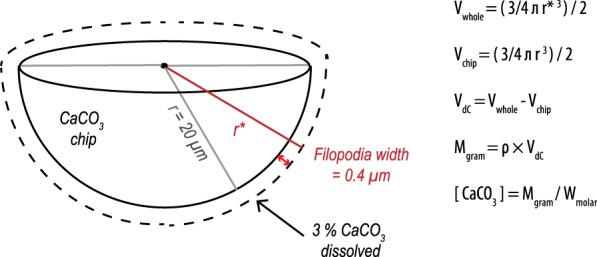
Chip schematic where V_dC_ is the volume of dissolved CaCO_3_, V_whole_ is the volume of the chip plus the volume of dissolved CaCO_3_, V_chip_ is the volume of the chip, M_gram_ is the mass of CaCO_3_ dissolved, ρ is the density of aragonite and W_molar_ the molecular mass of CaCO_3_.

Assuming vesicles have a pH of ~5, the concentration of protons in a given number of vesicles with an estimated volume can be translated to the maximum volume of CaCO_3_ that can be dissolved (Fig. 6). To do so we assume that the chip can be approximated by half of a sphere, with a diameter of 40 µm (the approximate average of observed excavated pits; see e.g. Fig. 4), the filopodia’s width is on average 0.4 µm and 3% of the chip is dissolved^9^. Based on these parameters, we estimate the amount of CaCO_3_ dissolved for each individual chip to equal 2.8 × 10^−11^ mol. The [H^+^] present in each vesicle of pH 5 is equal to 1 × 10^−5^ ml/L. Assuming vesicles take up the entire volume of the filopodia and each vesicle has a volume of 0.01 µm3 (diameter = 0.3 µm and a length equalling the radius of the semi sphere), a total of ~10^5^ vesicles can be squeezed in the filopodia surrounding the chip. These vesicles can therefore only provide 1 × 10^−17^ ml of protons for dissolution at one time. However, this number is already an overestimation as filopodia form intricate networks around chips and do not cover the entirety of the chip walls. In addition, although Fig. 5 depicts tightly packed vesicles within the filopodia, they do not appear to be filling the complete volume of the filopodia. If the etching process is completely achieved by exocytosis of low-pH vesicles, a continuous influx of vesicles and/or a constant replacement of filopodia and etching cells would be necessary. A single delivery of an acidic fluid would dissolve some CaCO_3_, but also be neutralized by the release of carbonate ions. Therefore, the low pH values observed at the etching sites need to be maintained over time and likely require active pumping and a continuous flux of protons.

### Conceptual model for sponge excavation of calcium carbonates

The overall observations and interpretations from the present study form the basis of an improved conceptual model for sponge bioerosion. Our results clearly demonstrate that a decrease in pH is actively created at the sponge/substrate interface. The high [H^+^] at this site is achieved through delivery of low-pH vesicles by the etching cells (Fig. 5) and leads to dissolution of the CaCO_3_ (here calcite) and subsequent disintegration of the filopodia. Dissolution of calcite neutralizes the released protons and elevates pH, after which the increased concentrations of Ca^2+^ and HCO_3_^−^ are removed before new acidic vesicles are emptied at the sponge-calcite interface. The manner and the speed in which Ca^2+^ and HCO_3_^−^ ions are transported away from the site of dissolution will inherently determine the speed at which CaCO_3_ can be dissolved. However, these processes remain to be investigated. Research on euendolithic phototrophic cyanobacteria demonstrated that microbial excavation is achieved through active intracellular Ca^2+^ transport^31^. Calcium chelators and a suite of proteins are known to bind calcium, and could hence be the origin of an active calcium flux from the substrate into the sponge’s cytoplasm. The protein proteoglycan is known to play a key role in the aggregation of dissociated sponge cells where calcium mediates the process by acting as an intracellular messenger^32^. When studying secondary metabolites, Sullivan and Faulkner^20^ demonstrated the presence of calcium chelators in *Siphonodictyon coralliphagum*^21^. Various siphonodictyals of this sponge were able to bind calcium ions and lower their activity in the solution. The authors displayed the potential reaction as a cycle in which the chelator molecule releases H^+^ and receives calcium ions at the site of dissolution, then releases calcium into the water column in exchange for a new H^+^ ion. In this pathway, a lowering of pH at the sponge/substrate interface must occur and this process would be affected by changes in seawater conditions such as an increase in *p*CO_2_^12,33^. However, the mechanism involved in transporting ions from the site of active dissolution to the water column remains unclear. Guida & Garcia-Pichel^31^ showed that long range transport of Ca^2+^ is mediated by calcium ATPases or channels through cells. A specialised cell was identified accumulating calcium at concentrations more than 500-fold compared to concentrations found in other cyanobacteria. The authors suggest that these cells permit fast calcium flow at nontoxic concentrations through undifferentiated cells by providing temporary storage for excess calcium before final excretion to the outside medium.

Calcium is known to play an important role for tissue contraction in marine sponges where cell to cell communication is used for tissue coordination^34,35^. The network of muscle-like cells that was observed on the calcite after removal of the “mother sponge” (Fig. 2) may provide a pathway for the intracellular transport of calcium ions out of the sponge and into the water column, similar to how muscle cells contract when triggered by an increase in intracellular Ca^2+^.

**Figure 7:**
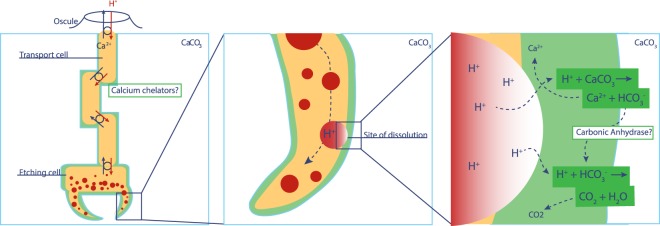
Working model for chemical dissolution of calcium carbonate by an excavating sponge. Protons are received in exchange for calcium ions (potentially via calcium chelators) at the water sponge interface. Protons are moved through the sponge via the transport cells until reaching the etching cells. They are then incorporated in vesicles (pH 4 to 5), which travel towards filopodia. Subsequently, the vesicles discharge their content through exocytosis, which leads simultaneously to the dissolution of calcium carbonate and disintegration of the etching cell. The new space is then rapidly colonised by new filopodia and so on. Newly freed bicarbonate ions are converted to CO_2_, a reaction made faster with carbonic anhydrase enzymes, which passively diffuses out of the sponge.

We propose a working model for sponge dissolution of calcium carbonate (Fig. 7) where protons are received in exchange for calcium ions (potentially via calcium chelators) at the water-sponge interface. Protons are moved through the sponge via the transport cells until reaching the etching cells. They are then incorporated in vesicles (pH ~5) that travel towards filopodia. Subsequently, the vesicles discharge their contents through exocytosis, which leads simultaneously to the dissolution of calcium carbonate and disintegration of the etching cell. The new space is then rapidly colonised by new filopodia, and the process continues. An efficient way to flush out HCO_3_^−^ would be to convert it to carbon dioxide so it can simply diffuse out of the sponge. Carbonic anhydrase enzymes have been located in great number around etching processes^8^ and may accelerate this conversion.

## Conclusion

These results show that CaCO_3_ dissolution by the excavating sponge *Cliona varians* is associated with the production of low pH vesicles in the filopodia of etching cells responsible for chip formation. These vesicles are transported within the etching cells towards the site of dissolution where they are emptied and create undersaturated conditions. The resulting high concentration of calcium at the sponge-calcite interface is hypothesized to be reduced by transport of calcium ions away from the site of dissolution, possibly directly balancing the flux of protons towards this site. The cells that connect the etching cells to the rest of the sponge tissue have a distinct morphology that is in line with transport of Ca^2+^ away from the site of dissolution. These findings reveal for the first time the basic mechanism by which excavating sponges dissolve calcium carbonate: the dependency of these sponges on the production of protons for CaCO_3_ dissolution may provide an explanation for the observed positive relationship between seawater *p*CO_2_ and dissolution rates^12–14,33^.

## Materials and Methods

### Sample collection and growth conditions

**Figure 8:**
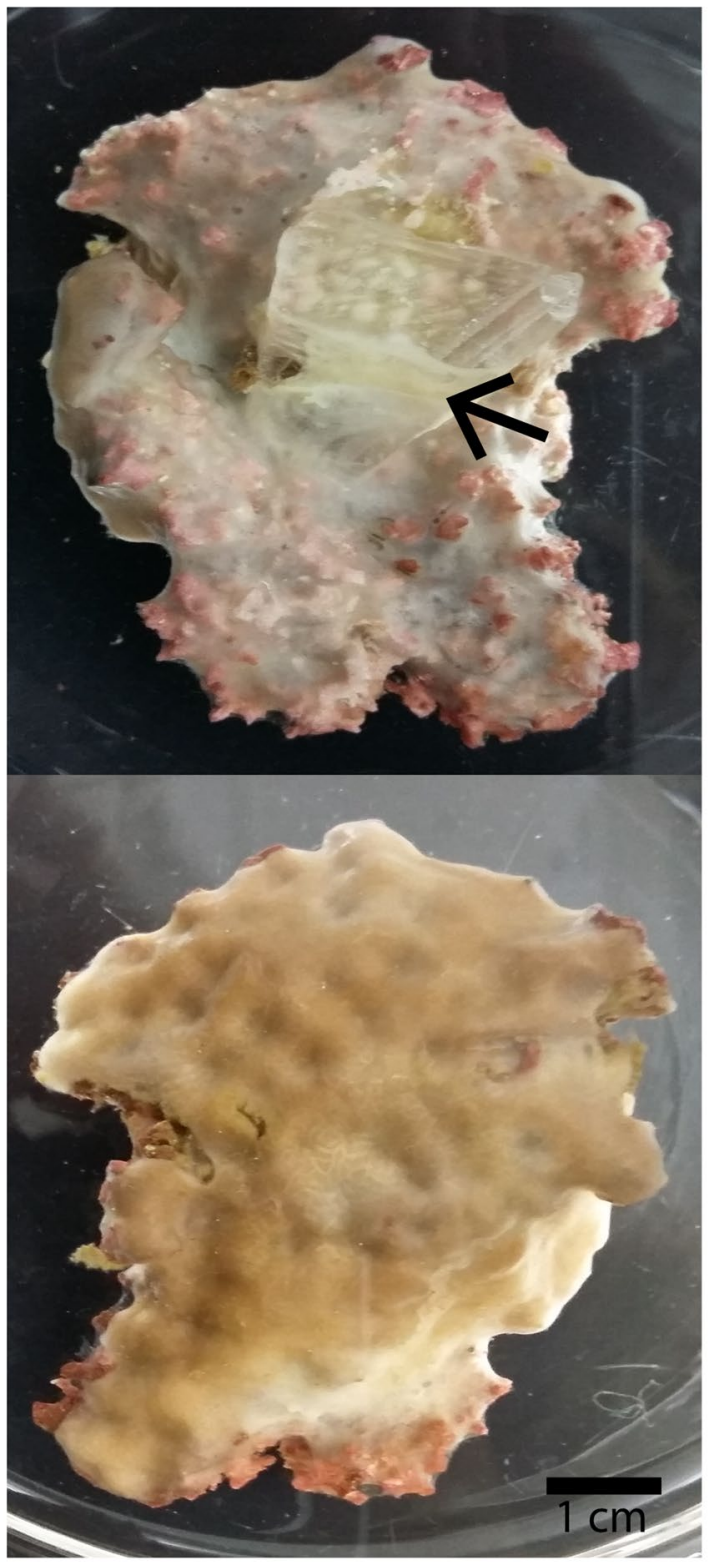
*Cliona varians* sample viewed from top (upper panel) and bottom (lower panel). Sponge has attached (arrow) to the piece of Iceland spar placed on its lower side.

Samples from 5 different *C*. *varians* sponges (Fig. 8) were collected off Summerland Key in the Florida Keys using a hammer and a chisel. Sponge samples (n = 30) were placed in flow-through seawater raceways at the Mote Tropical Marine Laboratory in Summerland Key, attached to pieces of Iceland spar (3 to 4 cm^3^) with plastic tie wraps, and subjected to a natural diurnal light cycle. They were kept in the flow-through raceways for one month to allow the cut surfaces to heal and attach to the Iceland spar. They were then transferred to Harbor Branch Oceanographic Institute (HBOI) at Florida Atlantic University, where they were placed in a recirculating seawater raceway in the aquaculture facility for 3 weeks before the experiment was started.

### Specimen preparation and staining

Pieces of calcite (Iceland spar) in early stages of sponge bioerosion were detached from the ‘mother sponge’ (Fig. 1) and broken into smaller thin fragments with surface areas ranging from 0.10 to 0.25 cm^2^ and with thickness of 0.1 to 0.2 cm. For each calcite piece, ~10 fragments were collected and stained following a derived protocol for pHrodo™ Red and Green AM Intracellular pH Indicators based on protocols developed by the manufacturer (Life Technologies). This involved dilution of 10 µL of stain into 100 µL of PowerLoad concentrate, dilution of the resulting solution into 10 mL of filtered seawater and incubation of the sponge fragments in 2 ml of this solution for 1 hour. Finally, the sponge/calcite fragments were rinsed in filtered seawater, placed in a 24-well plate and immediately imaged.

### Fluorescence Microscopy and Image Analysis

Fluorescence and transmitted light imaging was performed on an Invitrogen ^TM^ EVOS ^TM^ FL Auto Imaging System. Fluorescence emission intensity was measured after illuminating the incubated tissue with different excitation wavelengths. The red channel of emission in all images represents pH and was obtained using the Texas Red light cube with excitation and emission maxima around 585 and 624 nm, respectively. The commercial pHrodo™ indicator and Intracellular pH Calibration Buffer Kit (Thermofisher) were used for intracellular pH determination and pH calibration, respectively.

#### pHrodo™ Red Intracellular pH imaging

To assess intracellular pH, the fluorogenic intracellular pHrodo™ Red probe was utilized. pHrodo™ Red is weakly fluorescent at neutral pH but increasingly fluorescent as pH decreases. This reagent allows quantification of cellular cytosolic pH in the range of 4–9 with a pKa of ~6.5 and excitation/emission optima of 560/585 nm. To obtain absolute pH values, a pH calibration curve was produced using an Intracellular pH Calibration Buffer Kit.

Modification of pHrodo™ with AM ester groups results in an uncharged molecule that can permeate cell membranes. Once inside the cell, the lipophilic blocking groups are cleaved by nonspecific esterases, resulting in a compound that is retained within the intracellular space.

#### Intracellular pH Calibration Buffer Kit

The Intracellular pH Calibration Buffer Kit enables the quantification of intracellular pH when used in conjunction with pHrodo™ Red AM. It includes four pH calibration buffers (pH 4.5, 5.5, 6.5, and 7.5), as well as valinomycin and nigericin, which are used to equilibrate the pH inside and outside of cells. After staining dissociated cells (1 × 10^6^ cells/ml) from *C*. *varians* with pHrodo™ Red AM and detemining the fluorescence intensity under experimental conditions, cells were re-suspended in one of the calibration buffers and fluorescence intensity was measured again. This was performed in duplicate for each buffer to produce a calibration curve allowing translation of the sponge’s fluorescent images into pictures showing the internal pH distribution.

### Image processing for the calibration curve

**Figure 9:**
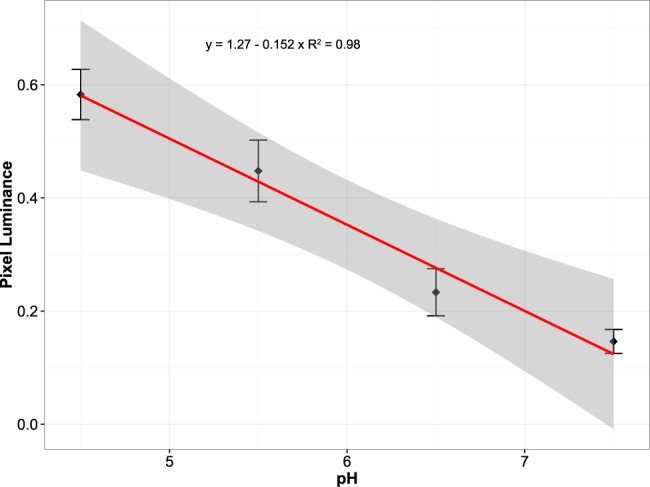
Calibration curve for intracellular pH in *C*. *varians* pixel luminescence intensity after removal of 1^st^ and 4^th^ quartiles. Error bars represent standard deviation derived from the variability in all pixels for the images taken. Uncertainty (grey envelope) surrounding the linear regression curve represents the 95% confidence intervals.

The calibration curve was produced using the programming environment R.3.4.3 (R Core Team, 2017). Each image is imported in R as a pixel matrix. Each given pixel has an intensity value between 0 and 1, corresponding to a luminosity scale from dark to light. Each image was corrected for background noise and excess luminescence due to potential leakage of the buffers out of the cells and superposition of the fluorescence signal. This was achieved by approximately removing the 1^st^ and 4^th^ quartile of the data, corresponding to 43% of the pixels from the original image (the removal of the data’s 4^st^ quartile led to an overestimation of pixels belonging to excess luminescence and was corrected for visually). The average pixel luminescence from each image was subsequently used to generate a calibration curve showing a significant linear relationship (R^2^ = 0.98, p < 0.0001) between intracellular pH and relative pixel fluorescence (and its variability: Fig. 9).

## Data Availability

The datasets generated during and/or analysed during the current study are available from the corresponding author on request.
